# Primary Cilia Are Critical Regulators of White Adipose Tissue Expansion

**DOI:** 10.3389/fphys.2021.769367

**Published:** 2021-10-25

**Authors:** Keren I. Hilgendorf

**Affiliations:** Department of Biochemistry, University of Utah School of Medicine, Salt Lake City, UT, United States

**Keywords:** primary cilia, diabetes, adipogenesis, signaling, obesity

## Abstract

The primary cilium is a microtubule-based cellular protrusion found on most mammalian cell types in diverse tissues. It functions as a cellular antenna to sense and transduce a broad range of signals, including odorants, light, mechanical stimuli, and chemical ligands. This diversity in signals requires cilia to display a context and cell type-specific repertoire of receptors. Recently, primary cilia have emerged as critical regulators of metabolism. The importance of primary cilia in metabolic disease is highlighted by the clinical features of human genetic disorders with dysfunctional ciliary signaling, which include obesity and diabetes. This review summarizes the current literature on the role of primary cilia in metabolic disease, focusing on the importance of primary cilia in directing white adipose tissue expansion during obesity.

## Introduction

White adipose tissue expands in response to caloric imbalance both by generating more adipocytes via *de novo* adipogenesis (hyperplasia) and by storing more fat in existing adipocytes (hypertrophy) (Haczeyni et al., 2018). The relative contribution of these two mechanisms of expansion has a profound effect on metabolic health, and this is independent of body mass index (BMI) (Ghaben and Scherer, 2019; Vishvanath and Gupta, 2019). Specifically, patients with metabolic disorders tend to have hypertrophic adipocytes, which in turn is linked to tissue hypoxia, inflammation, and fibrosis. In contrast, white adipose tissue containing smaller adipocytes, even if present in greater number, is associated with a healthy metabolic profile (Ye et al., 2021). Notably, mechanisms that promote adipogenesis have been shown to drive healthy white adipose tissue expansion in response to excess nutrients (Shao et al., 2018). Identifying ligands and signaling pathways that can trigger adipogenesis thus represent a therapeutic avenue to limit the pathogenic consequences of obesity.

The primary cilium is a sensory organelle that is highly conserved throughout eukaryotic evolution and found on most mammalian cells (Goetz and Anderson, 2010). All cells in the human body are either ciliated or derived from a ciliated stem cell (Yanardag and Pugacheva, 2021). Cilia are critical to human development and physiology by organizing signal transduction pathways such as hedgehog signaling in vertebrate cells (Kopinke et al., 2021). Consistent with the near ubiquitous presence and function of cilia in cells throughout the human body, a heterogeneous group of human genetic disorders with dysfunctional primary cilia (ciliopathies) manifest with a broad range of clinical features, including polydactyly, cysts in the kidney and liver, retinal degeneration, learning disabilities, obesity, and diabetes (Reiter and Leroux, 2017).

Understanding the function of primary cilia in diverse tissues can provide powerful insight into metabolic disease: Primary cilia on POMC (proopiomelanocortin) and AGRP (agouti-related protein) neurons in the arcuate nucleus of the hypothalamus are critical in the regulation of food intake and satiety sensing, and ciliary dysfunction in these neurons results in obesity due to excessive eating (hyperphagia) (Engle et al., 2020). In peripheral tissues, primary cilia on pancreatic islet cells regulate glucose homeostasis, primary cilia on cholangiocytes in the bile duct regulate bicarbonate secretion, and primary cilia on pre-adipocytes regulate adipogenesis (Gerdes et al., 2014; Mansini et al., 2018; Hilgendorf et al., 2019; Volta et al., 2019; Hughes et al., 2020; Wu et al., 2021). This review focuses on recent advances in our understanding of how the primary cilium can organize both pro- and anti-adipogenic signaling pathways during adipose tissue expansion in obesity.

## The Primary Cilium—The Cell’s Antenna

### Structure and Molecular Composition of Primary Cilia

**FIGURE 1:**
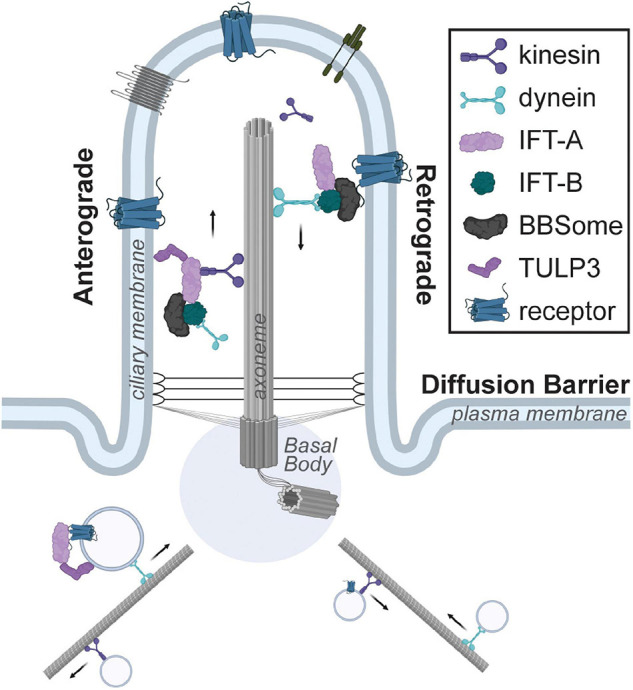
Schematic of the primary cilium. The cilium is composed of a microtubule-based axoneme and the surrounding ciliary membrane, which is highly enriched for receptors. The intraflagellar transport system couples to kinesin or dynein motors for anterograde or retrograde transport within the cilium. Trafficking of ciliary cargo such as receptors into or out of the primary cilium is tightly regulated and requires adaptor proteins such as TULP3 or the BBSome complex, respectively.

There are two broad classes of cilia. Motile cilia/flagella are found on specialized cells. This includes sperm, where the flagellum enables motility, and ependymal cells, where multiple motile cilia generate directional flow of cerebrospinal fluid (Mitchison and Valente, 2017). In contrast, most other mammalian cells contain a single, immotile primary cilium that is 2–10 μm in length (Nachury and Mick, 2019). This primary cilium is comprised of a microtubule-based axoneme and a surrounding ciliary membrane (Figure 1). The axoneme consists of nine outer microtubule doublets arranged in a 9 + 0 fashion and is nucleated by the basal body (Fisch and Dupuis-Williams, 2011). The basal body is composed of the mother centriole and pericentriolar material, and it is anchored to the cell membrane by distal appendages. Since centrosomes are required for the formation of both the mitotic spindle and the primary cilium, the biogenesis of the primary cilium is cell cycle-dependent, with ciliary disassembly occurring prior to mitosis and reassembly occurring during the G_1_ or G_0_ phase of the cell cycle (Ford et al., 2018; Ho et al., 2020). Within the primary cilium, the intraflagellar transport (IFT) machinery couples to the microtubule motor proteins kinesin-2 or dynein-2 to transport proteins along the axoneme in an anterograde or retrograde direction, respectively.

The ciliary membrane is contiguous with the plasma membrane. To generate a unique and compartmentalized signaling organelle, the transition zone at the base of the cilium functions as a diffusion barrier (Malicki and Johnson, 2017). The ciliary membrane has a unique lipid composition enriched for the phosphoinositide PI(4)P, rather than the PI(4,5)P_2_ typically found in the plasma membrane, and this is generated via the ciliary localization of the ciliopathy-associated phosphoinositide 5-phosphatase INPP5E (Chavez et al., 2015; Garcia-Gonzalo et al., 2015; Jensen et al., 2015). Similarly, a high ciliary concentration of calcium channels generates an approximately sevenfold higher resting calcium concentration in the cilium compared to the cytoplasm (DeCaen et al., 2013; Delling et al., 2013). The localized concentration of cyclic AMP (cAMP) may also be elevated in the primary cilium compared to the cytoplasm in some cell types (Moore et al., 2016; Jiang et al., 2019; Truong et al., 2021). Finally, the ciliary membrane is highly enriched for receptors and its molecular composition varies according to the cell type and cellular signaling status (Hilgendorf et al., 2016). Numerous mechanisms regulate trafficking of ciliary cargo into and out of the primary cilium (Nachury et al., 2007; Mukhopadhyay et al., 2010; Wright et al., 2011). Most clinical features of ciliopathies are due to genetic mutations deregulating ciliary entry and exit, cargo trafficking, and signaling.

### Ciliary Signal Transduction Pathways

Numerous receptors localize to the primary cilium, including several receptors critical for energy homeostasis and metabolic health (Table 1). Depending on the cell and tissue context, these signaling pathways organized by the primary cilium can have both positive and negative effects on energy homeostasis. Intriguingly, some receptors can localize to and signal from both the primary cilium and the plasma membrane. We do not fully understand why ciliary localization is required for some receptors and in some cellular contexts to signal. One possible explanations is that the increased surface to volume ratio of the primary cilium and the unique ciliary signaling environment may increase the sensitivity of a signaling pathway to a ligand. The distinct composition of ciliary proteins and metabolites may also allow receptors to activate different downstream effector proteins in the cilium vs. the plasma membrane. Finally, the primary cilium may enable the sensing and integration of multiple simultaneous signals into common ciliary second messengers or effector proteins to elicit a binary cellular response (Hilgendorf et al., 2016; Malicki and Johnson, 2017; Nachury and Mick, 2019).

**TABLE 1:** List of receptors relevant to energy homeostasis and capable of localizing to the primary cilium.

**Receptor**	**References for ciliary localization**	**Cell and tissue contexts described for ciliary localization *(overexpressed* and endogenous)**	**Ligand(s)**	**Function related to energy homeostasis**
DRD1	Marley and von Zastrow, 2010; Domire et al., 2011	*NIH3T3, IMCD3*, several regions of brain including primary striatal neurons and amygdala	Dopamine	Feeding
EP4/PTGER4	Jin et al., 2014; Wu et al., 2021	RPE-1, IMCD3, Min6, aTC9, pancreatic beta and alpha cells	Prostaglandin E2	Glucagon and insulin secretion
FFAR4/GPR120	Hilgendorf et al., 2019; Wu et al., 2021	3T3-L1, white adipose tissue, Min6, αTC9, pancreatic beta and alpha cells	Omega-3 fatty acids	Adipogenesis; glucagon and insulin secretion
GALR2	Loktev and Jackson, 2013	*RPE-1*	Galanin	Feeding
GALR3	Loktev and Jackson, 2013	*RPE-1*, cultured hypothalamic neurons, hypothalamus section	Galanin	feeding
GPR83	Loktev and Jackson, 2013	*RPE-1*, several regions of brain, e.g., olfactory tubercle, nucleus accumbens	Orphan	Feeding
HTR6	Hamon et al., 1999; Brailov et al., 2000	Several regions of brain including nucleus accumbens and olfactory tubercles	Serotonin	Feeding
IGF-1R	Zhu et al., 2009; Dalbay et al., 2015	3T3-L1, MSCs	IGF-1, insulin	Multiple including adipogenesis
Insulin Receptor (IR isoform A only)	Gerdes et al., 2014	Min6, pancreatic beta cells	Insulin	Beta cell insulin secretion (IR-A is not expressed in mature adipocytes
KISSR1	Koemeter-Cox et al., 2014	*IMCD3*, medial hypothalamus	Kisspeptin	Physical activity, energy expenditure
MC4R	Siljee et al., 2018	*MEF*, *RPE-1*, *IMCD3*, subset of hypothalamic neurons	Melanocyte stimulating hormone	Feeding
MCHR1	Berbari et al., 2008	*IMCD3*, nucleus accumbens	Melanin-concentrating hormone	Feeding
NMUR1	Omori et al., 2015	*NIH3T3*	Neuromedin U	Feeding
NPY2R	Loktev and Jackson, 2013; Omori et al., 2015	*NIH3T3*, *RPE-1*, cultured hypothalamic neurons, hypothalamus section (arcuate nucleus)	Neuropeptides NPY, peptide YY	Feeding
NPY5R	Loktev and Jackson, 2013	RPE-1, cultured hypothalamic neurons, hypothalamus section	Neuropeptides NPY, peptide YY	Feeding
PRLHR	Omori et al., 2015	*NIH3T3*, third ventricle mouse brain	Prolactin-releasing hormone	Feeding
PTCH1	Rohatgi et al., 2007	*MEFs*, NIH3T3, mouse embryo mesoderm cells	Hedgehog	Multiple including MSC commitment
QRFPR	Loktev and Jackson, 2013	*RPE-1*, cultured hypothalamic neurons, hypothalamus section	Neuropeptide QRFP	Feeding
SMO	Corbit et al., 2005	*MDCK*; MEFs, IMCD3, nodal cells	Oxysterols	Multiple including MSC commitment
SSTR3	Handel et al., 1999; Iwanaga et al., 2011	Several regions of brain including hypothalamus, amygdala and cerebellum; pancreatic islets; anterior pituitary	Somatostatin	Feeding
TGR5	Keitel et al., 2010	Isolated cholangiocytes, liver sections	Bile acid	Bile composition
P2YR12	Masyuk et al., 2008	Rat liver section (cholangiocytes)	ADP	Bile composition

Most ciliary receptors identified to-date are G protein-coupled receptors (GPCRs) (Wachten and Mick, 2021). Trafficking of GPCRs into and out of the primary cilium is tightly regulated. The TUBBY family of proteins is required for trafficking GPCRs to the primary cilium (Mukhopadhyay et al., 2010; Sun et al., 2012). Expression of TUBBY family members is tissue-specific, with TULP3 being expressed most broadly (Badgandi et al., 2017). Another multi-protein complex, the BBSome, is required for β-arrestin2 mediated removal of GPCRs out of the primary cilium (Lechtreck et al., 2009; Ye et al., 2018). Since genes mutated in Bardet-Biedl syndrome encode the BBSome, the clinical features of this ciliopathy are likely a result of aberrant ciliary receptor composition (Nachury et al., 2007). Together, these two protein families are integral to the generation of cell type-specific repertoires of ciliary GPCRs appropriate for a particular biological context (Figure 1). However, we do not yet understand how this cell and context-specific trafficking of GPCRs is achieved.

## Ciliopathies and Metabolic Disease

Genetic disorders affecting the biogenesis and maintenance of the cilium, known collectively as ciliopathies, serve as powerful tools to discover new drivers of human disease (Wheway et al., 2018). A subset of ciliopathies is associated with obesity: Alström syndrome (ALMS, OMIM #203800) and Bardet-Biedl syndrome (BBS, OMIM #209900). ALMS is caused by mutations in the *ALMS1* gene (Collin et al., 2002; Hearn et al., 2002). ALMS1 localizes to the centrosome, including the basal body in ciliated cells (Knorz et al., 2010). While the molecular function of ALMS1 has not been fully elucidated, ALMS1 is thought to regulate ciliary signaling and transport of ciliary cargo (Li et al., 2007; Jagger et al., 2011). BBS is caused by mutations in one of 22 genes and the majority of BBS genes encode proteins that together form or are associated with a large complex called the BBSome (Nachury et al., 2007). The BBSome is required for transporting a subset of GPCRs out of the primary cilium. Despite the similarities of BBS and ALMS with regard to obesity and ciliary dysfunction, the two disorders present notable differences with regard to metabolic health: ALMS patients are moderately obese with extreme insulin resistance and increased incidence of early onset type 2 diabetes mellitus compared to patients matched for pubertal stage and body composition (Minton et al., 2006). In contrast, even though BBS patients are morbidly obese and have greater visceral adiposity, patients have better glucose tolerance than BMI-matched control subjects (Beales et al., 1999; Feuillan et al., 2011; Marion et al., 2012). This disparity illustrates the complexity of ciliary signaling pathways, with primary cilia regulating numerous cellular processes in diverse tissues. Since the main focus of this review is to discuss the role of cilia in adipose tissue, the reader is referred to a more detailed review for the role of cilia in other contexts including in hypothalamic neurons to regulate satiety sensing (Engle et al., 2020).

### Alström Syndrome

Patients with Alström syndrome and ALMS1 mutant mice are obese, but with increased insulin resistance that is disproportionate to body weight, adiposity, and fat distribution (Gathercole et al., 2016; Han et al., 2018). Obesity develops early during childhood, along with hypertriglyceridemia, hyperinsulinemia, extreme insulin resistance, and the development of type 2 diabetes mellitus with a mean age of onset at 16 years (Marshall et al., 2011). The BMI tends to normalize in older individuals, but insulin resistance continues to increase (Minton et al., 2006). Childhood hyperphagia and lower levels of physical activity may contribute to the development of obesity. However, while all ALMS mouse models recapitulate the clinical features of the disease, including obesity and insulin resistance, this is not driven by hyperphagia in most of these mouse models (Marshall et al., 2011). This suggests that ALMS1 functions in peripheral organs to regulate metabolic health, rather than in the central nervous system.

Patients with ALMS1 have increased adipocyte hypertrophy in subcutaneous white adipose tissue when compared to BMI-matched control subjects (Geberhiwot et al., 2021). Molecularly, depletion of ALMS1 in pre-adipocytes inhibits adipogenesis and depletion of ALMS1 in mature adipocytes impairs glucose uptake via an insulin signaling-independent mechanism (Huang-Doran and Semple, 2010; Favaretto et al., 2014). ALMS1 mutant mice present with hyperglycemia, insulin resistance and hyperleptinemia prior to the development of obesity (Geberhiwot et al., 2021). Together, this argues that mutations in ALMS1 cause adipose tissue dysfunction, which in turn drives insulin resistance, type 2 diabetes mellitus, and obesity in ALMS patients. In support of this model, Geberhiwot et al. (2021) recently generated a mouse model re-expressing ALMS1 in the adipose tissue of mutant mice using an *Adipo*-CRE allele. Remarkably, ALMS1 expression in adipose tissue completely rescued obesity, adipocyte hypertrophy, glucose tolerance, and insulin sensitivity. Of note, both fetal pre-adipocytes and mature adipocytes express adiponectin and hence *wild-type* ALMS1 in this mouse model (Hong et al., 2015). Further investigation using a CRE recombinase that is specific to mature adipocytes may ascertain the relative contribution of ALMS1 function in adipogenesis vs. adipocyte glucose uptake. Taken together, these data show that ALMS1 mutation causes adipose tissue dysfunction, leading to the development of obesity, extreme insulin resistance, and early-onset type 2 diabetes mellitus in ALMS patients.

### Bardet-Biedl Syndrome

Mouse models of BBS are obese due to hyperphagia, decreased locomotor activity, and hyperleptinemia (Rahmouni et al., 2008). This reproduces the clinical features of human patients, including evidence that BBS patients have lower levels of physical activity when compared to BMI-matched control subjects (Grace et al., 2003). In mouse knockout models, leptin resistance precedes the development of obesity, suggesting that dysfunctional satiety sensing leads to obesity in BBS (Rahmouni et al., 2008). Notably, pair-feeding (matching the amount of food consumed) normalizes the body weight of BBS knockout mice. Yet despite the normalized body weight, BBS knockout mice still present with increased adiposity (Rahmouni et al., 2008). This suggests that the BBSome has additional roles outside the central nervous system, including in adipose tissue.

Molecularly, depletion of BBS12 in pre-adipocytes promotes adipogenesis (Marion et al., 2009). Visceral adipocytes in *Bbs12* knockout mice are heterogeneous in size and visceral adipose tissue expansion in *Bbs12* knockout mice is driven by both hyperplasia and adipocyte hypertrophy (Marion et al., 2012). As described above, excessive adipocyte hypertrophy is linked to the development of type 2 diabetes mellitus, while hyperplasia in adipose tissue is generally considered metabolically healthy (Ghaben and Scherer, 2019; Vishvanath and Gupta, 2019). Consistent with this model, *Bbs12* knockout mice have improved, rather than diminished insulin and glucose tolerance compared to lean littermates despite increased body weight and adiposity (Marion et al., 2012). Similarly, *Bbs12* knockout mice on a high fat diet are more obese, but have lower blood glucose levels and white adipose tissue inflammation compared to control littermates. Together, this argues that the BBSome can regulate both food intake and adipogenesis, such that loss-of-function mutations in BBS genes cause hyperphagia-driven obesity as well as metabolically healthy, hyperplastic white adipose tissue expansion.

Thus, while both ALMS and BBS patients are obese, this is driven by distinct functions of the primary cilium, either exclusively in pre-adipocytes (ALMS) or in multiple tissues (BBS), resulting in dramatic differences regarding metabolic health. Of note, some studies have described higher, rather than lower incidence of metabolic disease in BBS patients when normalized to BMI-matched control patients, and this disparity may be explained by the noted misdiagnoses of ALMS patients as BBS patients in cases where categorization was purely based on clinical features (Aliferis et al., 2012).

## Primary Cilia Direct How White Adipose Tissue Expands

All characterized stem and progenitor cells in the human body are ciliated (Yanardag and Pugacheva, 2021). This includes the embryonic stem cell, mesenchymal stem cells (MSCs), and all committed progenitor cells in the mesenchymal lineages giving rise to adipose tissue, muscle, bone, and cartilage. Thus, the primary cilium can be used as a marker of stem cells *in vivo*. Moreover, numerous studies have shown that stem cell ciliation is also critical for differentiation (Tummala et al., 2010; Kopinke et al., 2017; Lyu and Zhou, 2017). Since differentiation is regulated by tissue-specific ligands, this suggests that stem and progenitor cells in different tissues express context-specific, ciliary receptors to sense ligands and regulate stem cell fate.

### The Primary Cilium Is a Biomarker for Pre-adipocytes in White Adipose Tissue

MSCs and committed pre-adipocytes, pre-osteoblasts, pre-chondrocytes, and muscle stem cells are ciliated (Marion et al., 2009; Jaafar Marican et al., 2016; Yuan and Yang, 2016). Specifically, we recently showed that approximately 80% of isolated murine pre-adipocytes (Lin^–^ CD34^+^ CD29^+^ SCA1^+^) are ciliated (Hilgendorf et al., 2019). Using whole-mount imaging on a transgenic mouse model with fluorescently marked cilia, we also showed that approximately 30% of all perivascular cells in both subcutaneous (inguinal) and visceral (epididymal, perirenal, and mesenteric) white adipose tissue are ciliated *in vivo*. Similarly, we confirmed that PDGFRα-lineage traced perivascular cells in murine white adipose tissue are ciliated *in vivo*. Finally, we showed that ciliated perivascular pre-adipocytes are activated to reenter the cell cycle in response to high fat diet as determined by BrdU incorporation. Thus, we propose that the primary cilium can be used as a biomarker to visualize the location, abundance, and adipogenic responsiveness of pre-adipocytes in white adipose tissue. Notably, approximately 75% of isolated human pre-adipocytes are also uniformly ciliated (Marion et al., 2009; Forcioli-Conti et al., 2015).

The 3T3-L1 pre-adipocyte cell line as well as isolated primary human and murine pre-adipocytes *in vitro* become uniformly ciliated upon reaching confluency and becoming growth-arrested (Zhu et al., 2009). Mature, lipid-laden adipocytes are not ciliated (Marion et al., 2009; Forcioli-Conti et al., 2015; Hilgendorf et al., 2019). Proliferating pre-adipocytes are also not ciliated, consistent with the cell cycle-dependent biogenesis of primary cilia. Growth-arrest is required for pre-adipocytes to differentiate *in vitro* (Ailhaud et al., 1989). This suggests that the pre-adipocyte primary cilium functions to regulate initiation of adipogenesis.

### Dynamic Remodeling of Ciliary Receptors During Adipogenesis Enables Multi-Functionality of the Primary Cilium

**FIGURE 2:**
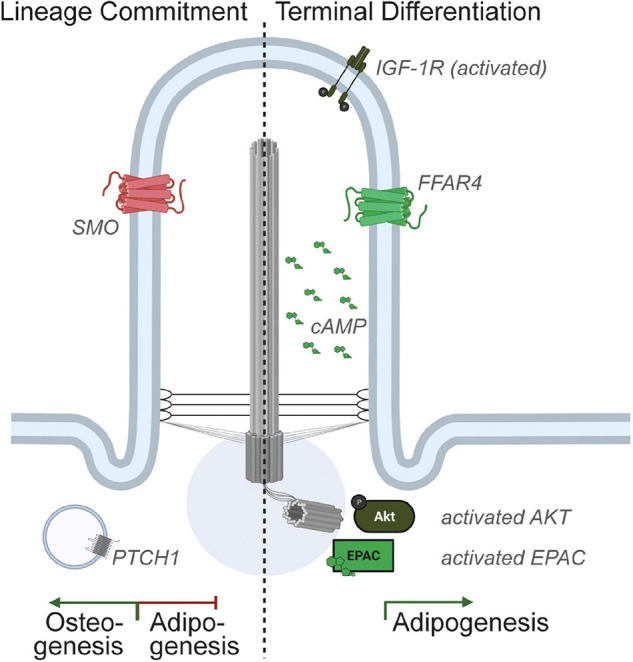
Ciliary signaling in pre-adipocytes. Multiple receptors localize to the pre-adipocyte primary cilium and activation of ciliary signaling pathways can either promote or inhibit adipogenesis. Specifically, Hedgehog signaling has been shown to inhibit MSC commitment to adipogenesis. In contrast, the ciliary IGF-1 receptor and ciliary FFAR4/GPR120 are expressed in committed pre-adipocytes, and activation of these ciliary signaling pathways promotes adipogenesis.

Genetic manipulations of MSCs and pre-adipocytes have revealed that the primary cilium can both inhibit and promote adipogenesis. Specifically, depletion of BBS12, FBF1, or PKD1 promotes adipogenesis, while depletion of ALMS1, KIF3A, or IFT88 inhibits adipogenesis (Marion et al., 2009, 2012; Zhu et al., 2009; Huang-Doran and Semple, 2010; Qiu et al., 2010; Zhang et al., 2021). Notably, we recently showed that genetic ablation of ciliation specifically in pre-adipocytes of adult male and female mice, via the depletion of *Ift88* using a tamoxifen-inducible *Pdgfr*α-CreERT, completely prevented *in vivo* adipogenesis (Hilgendorf et al., 2019). We propose that the primary cilium functions to inhibit MSC commitment to the adipogenic lineage, but is required for adipogenesis *in vitro* and *in vivo*. The ciliary signaling pathways discovered to-date that mediate these pro- and anti-adipogenic functions are discussed below (Figure 2).

Cell biological findings support the hypothesis that the primary cilium is dynamic, showing that the morphology of the primary cilium changes during MSC commitment and during adipogenesis. Specifically, the relative prevalence of ciliation and the length of the primary cilium increases in isolated human MSCs exposed to adipogenic differentiation media (Dalbay et al., 2015; Forcioli-Conti et al., 2015). In contrast, exposure to osteogenic differentiation media results in a decrease in percent ciliation but increase in ciliary length, while exposure to chondrogenic differentiation media causes a decrease in percent ciliation and decrease in ciliary length (Dalbay et al., 2015). Further, ciliary length is maximal on Day 3 of MSC adipogenesis, before decreasing in length once again as the differentiating pre-adipocyte starts to accumulate lipid (Forcioli-Conti et al., 2015). Finally, the mature, lipid-laden adipocyte no longer has a primary cilium. The transient increase in ciliary length during adipogenesis is dependent on the IFT machinery and mediated by the presence of the glucocorticoid dexamethasone in the adipogenic differentiation media (Dalbay et al., 2015; Forcioli-Conti et al., 2015). Ciliary length is controlled by trafficking and recruitment of ciliary cargo (Kuhns et al., 2019).

We postulate that the molecular composition of the primary cilium, including ciliary receptors, dynamically changes during MSC commitment and adipogenesis. This ciliary remodeling would enable the primary cilium to sense and appropriately respond to extracellular signals. Of note, both ciliary and non-ciliary signaling can induce dynamic changes to the length of the primary cilium in both epithelial and mesenchymal cells, including PKA and MAPK-mediated ciliary lengthening (Besschetnova et al., 2010; Abdul-Majeed et al., 2012). This suggests that ciliary remodeling may be part of a positive feedback loop, such that the MSC primary cilium becomes more sensitive to adipogenic signals upon receiving sufficient signals to commit to the adipogenic lineage. Further ciliary remodeling may also occur during terminal differentiation.

### Ciliary Signaling Pathways Inhibiting Adipogenesis

The hedgehog signaling pathway is essential for both embryonic development and for adult tissue homeostasis, and the primary cilium is required for hedgehog signaling in vertebrate cells (Huangfu et al., 2003; Briscoe and Therond, 2013). Activation of the hedgehog signaling pathway inhibits adipogenesis of the multipotent MSC cell lines C3H/10T1/2 and ST2 via upregulation of osteogenic markers (Spinella-Jaegle et al., 2001; Suh et al., 2006). Thus, ciliary hedgehog signaling can regulate MSC commitment toward osteogenesis. Loss of primary cilia in this context results in de-repression and hence potentiation of adipogenesis.

Genetic alterations that change the ciliary receptor composition can promote adipogenesis. Specifically, depletion of BBS12 promotes adipogenesis in human primary MSCs and causes adipocyte hyperplasia in *Bbs12* knockout mice (Marion et al., 2009, 2012). Since the BBSome is required for ciliary removal of receptors, we postulate that loss of BBSome function results in ciliary remodeling in MSCs, which in turn results in increased adipogenic commitment and/or increased recruitment of MSCs to white adipose tissue. Similarly, mouse models deficient for *Fbf1*, which can regulate ciliary entry and exit by ensuring proper BBSome assembly, are obese with improved glucose and insulin tolerance, in part due to increased white adipose tissue hyperplasia and beiging (Zhang et al., 2021). Mouse models deficient for *Pkd1*, which encodes the protein polycystin-1 and is commonly mutated in autosomal dominant polycystic kidney disease, have skeletal abnormalities, and isolated MSCs and primary osteoblasts have a decreased osteogenic but increased adipogenic potential (Qiu et al., 2010). Depletion of the axonemal kinesin-2 subunit KIF3A, required for trafficking of ciliary cargo and hence for any ciliary remodeling, rescues the abnormal skeletal development in the *Pkd1^–/–^ Kif3a^–/–^* double mutant and leads to decreased adipogenesis. Together, this argues that mutations that lead to ciliary receptor remodeling affect MSC commitment to the adipogenic vs. osteogenic lineage.

Interestingly, while hedgehog signaling inhibits adipogenesis in white adipose tissue, it does not inhibit adipogenesis in brown fat (Pospisilik et al., 2010; Nosavanh et al., 2015). Instead, non-canonical hedgehog signaling promotes insulin-independent glucose uptake in brown adipose tissue (Teperino et al., 2012). This suggests that the ciliary receptor repertoire of progenitor cells differs in white compared to brown adipose tissue.

### Ciliary Signaling Pathways Promoting Adipogenesis

While the primary cilium can inhibit MSC commitment and hence adipogenesis, numerous studies in which the primary cilium was genetically ablated in committed pre-adipocytes unequivocally showed that primary cilia on pre-adipocytes are required for adipogenesis. Specifically, removal of the primary cilium via depletion of *Ift88* or *Kif3a*, genes required for the biogenesis and maintenance of primary cilia, in 3T3-L1 preadipocytes or *in vivo* in PDGFRα-lineage perivascular cells, dramatically inhibits adipogenesis (Zhu et al., 2009; Hilgendorf et al., 2019). Consistent with these data, ALMS1 depletion in 3T3-L1 pre-adipocytes inhibits adipogenesis and ALMS patients and mouse models present with adipocyte hypertrophy (Huang-Doran and Semple, 2010; Geberhiwot et al., 2021). Thus, pre-adipocyte primary cilia express ciliary receptors that can promote adipogenesis. To-date, two pro-adipogenic ciliary receptors have been identified.

Zhu et al. (2009) showed that the IGF-1 receptor localizes both to the primary cilium and the plasma membrane in growth-arrested 3T3-L1 pre-adipocytes. Intriguingly, ciliary IGF-1R was more sensitive than IGF-1R localized to the plasma membrane, with insulin stimulation activating the receptor (as determined by tyrosine phosphorylation) both faster and at lower concentrations of insulin. Downstream, insulin stimulation resulted in the accumulation of activated IRS1 and AKT at the basal body. Thus, the primary cilium may increase the sensitivity of pre-adipocytes to insulin, lowering the threshold at which insulin triggers adipogenesis *in vivo*. Another recent study showed that IGF-1R and AKT can also accumulate in caveolin-containing lipid rafts at the ciliary base upon insulin stimulation, and that this is cilia-dependent (Yamakawa et al., 2021). We do not yet understand why ciliary IGF-1R is more sensitive to insulin stimulation than IGF-1R localized to the plasma membrane.

Further, Dalbay et al. (2015) showed that IGF-1R also localizes to the primary cilium of MSCs induced to undergo adipogenesis. Ciliary recruitment of IGF-1R is IFT-dependent and concurrent with the transient elongation of the primary cilium in differentiating MSCs on Day 3 of MSC adipogenesis. At this same time-point, pre-adipocytes become less responsive to anti-adipogenic Sonic Hedgehog signaling (Forcioli-Conti et al., 2015). Together, this argues that the primary cilium is remodeled upon commitment to express ciliary IGF-1R, which increases the adipogenic potential of the committed pre-adipocyte. What regulates the recruitment of IGF-1R from the plasma membrane to the primary cilium upon lineage commitment remains to be elucidated.

Recently, we identified the presence of an additional pro-adipogenic, ciliary receptor in pre-adipocytes (Hilgendorf et al., 2019). Specifically, we discovered that depletion of TULP3 potently inhibited adipogenesis. TULP3 is an adaptor protein that is required for trafficking of GPCRs to the primary cilium. Subsequent screening for ciliary localization of GPCRs expressed in pre-adipocytes identified that the omega-3 fatty acid receptor FFAR4/GPR120 is ciliary in 3T3-L1 pre-adipocytes, primary isolated mouse and human pre-adipocytes, and pre-adipocytes *in vivo*. Activation of ciliary FFAR4 by the omega-3 fatty acid DHA (docosahexaenoic acid) triggers adipogenesis by activating ciliary cAMP, which in turn activates the guanine-nucleotide exchange factor EPAC. Further downstream, DHA promotes activation of the regulator of chromatin architecture CTCF and looping of enhancers to promoter sites of several adipogenic genes including *Cebp*α, inducing their transcription. Non-FFAR4 ligands, such as saturated and mono-unsaturated fatty acids, do not trigger adipogenesis. Notably, the importance of cAMP and EPAC to the initiation of adipogenesis is well established, as illustrated by the inclusion of the phosphodiesterase inhibitor 3-isobutyl-1-methylxanthine (IBMX) in the standard adipogenic cocktail (Petersen et al., 2008). The primary cilium and the discovery of ciliary FFAR4 now link a physiological ligand, dietary omega-3 fatty acids, to cAMP elevation in pre-adipocytes to promote adipogenesis.

Intriguingly, FFAR4, like IGF-1R, can localize to both the primary cilium and the plasma membrane. In mature adipocytes, which are not ciliated, activation of FFAR4 in the plasma membrane by omega-3 fatty acids promotes glucose uptake in a G_α__q_-dependent manner (Oh et al., 2010). This suggests that white adipose tissue can utilize the same ligand-receptor pair and respond to nutritional fluxes in dietary fatty acids by both activating pre-adipocytes (via ciliary FFAR4 and cAMP) and increasing glucose uptake in mature adipocytes (via plasma membrane-localized FFAR4 coupled to G_α__q_). We speculate that ciliary vs. plasma membrane localization of FFAR4 enables compartmentalized, differential G_α_ coupling. What regulates trafficking of FFAR4 to the primary cilium in pre-adipocytes remains to be elucidated.

Genetic ablation of cilia inhibits adipogenesis *in vitro* and *in vivo*, showing that one of more ciliary signaling pathways are required for adipogenesis. As described above, both ciliary IGF-1R and ciliary FFAR4 can promote adipogenesis and we do not yet understand their relative importance to this process. Moreover, additional ciliary signaling pathways may exist in pre-adipocytes, including anti-adipogenic ones. Recently, Cook et al. (2021) showed that the melanin concentrating hormone receptor MCHR1 is not only expressed in the primary cilium of hypothalamic neurons, but also in 3T3-L1 pre-adipocytes, and that MCH inhibits adipogenesis. Together, this suggests that the primary cilium may function like a decision center for the committed pre-adipocyte, sensing and integrating both pro- and anti-adipogenic signals to regulate adipogenesis and white adipose tissue expansion in obesity.

## Conclusion and Future Challenges

The primary cilium is a signaling hub for the cell. Ciliary signaling mechanisms are critical for energy homeostasis, and this is illustrated by the clinical features of two ciliopathies, Bardet-Biedl syndrome and Alström syndrome. However, primary cilia in different cell types and tissue contexts express a distinct repertoire of receptors. We are only starting to decipher which signaling pathways are organized by the primary cilium in which cells. We do not yet know the functional significance and molecular mechanisms underlying the regulated trafficking of ciliary receptors and signaling components to the primary cilium in certain cells, but not in all ciliated cells.

Mouse genetics show that the primary cilium inhibits commitment to the adipogenic lineage, but is required for adipogenesis of the committed pre-adipocyte. Further, the primary cilium of committed pre-adipocytes can organize both pro-and anti-adipogenic signaling pathways. We postulate that the primary cilium undergoes dynamic remodeling during lineage commitment and adipogenesis. Future investigation using proximity proteomics may unravel how the protein composition of the primary cilium varies according to cell type and differentiation status.

It is intriguing to consider that ciliary receptor composition or ciliary receptor sensitivity differ in distinct contexts associated with differential adipogenic potential, such as pre-adipocytes located in subcutaneous vs. visceral white adipose tissue, in men vs. women, young vs. old, and lean vs. obese individuals. Interestingly, Ritter et al. (2018) showed that human adipose-derived MSCs isolated from lean vs. obese female patients differ in both the prevalence of ciliation and ciliary length. Specifically, both subcutaneous and visceral pre-adipocytes from obese subjects had fewer and shorter primary cilia. This ciliation defect correlated with decreased adipogenic potential, decreased motility, and increased secretion of inflammatory cytokines. This suggests that ciliary shortening may be a pathogenic consequence of obesity. We do not yet know if changes to ciliary length alter the expression of ciliary receptors and thus may affect the ciliary, adipogenic signaling pathways described above. Future investigations will establish the temporal relationship between the ciliation defect and the adipogenesis defect, whether the ciliation defect of obese adipose-derived MSCs contributes to pathology, and if cilia are restored upon weight loss. Interestingly, addition of inhibitors against cilia-destabilizing kinases rescues ciliary length of obese adipose-derived mesenchymal stem cell and increases both their migratory and adipogenic capacity (Ritter et al., 2019).

Together, these studies suggest targeting of ciliary signaling pathways as a therapeutic strategy to increase the adipogenic potential of pre-adipocytes, including in obese white adipose tissue to promote hyperplastic white adipose tissue expansion and potentially limit the incidence and severity of metabolic disease. Further investigation is necessary to identify potential targets to trigger pro-adipogenic, ciliary signaling pathways directly. We postulate that the primary cilium may function as a decision center, integrating multiple signals to elicit the binary response of triggering adipogenesis. Molecularly, this may be achieved by reaching a threshold amount of second messengers such as calcium or cAMP in the primary cilium to activate adipogenic kinases and exchange factors, and further investigation using live-imaging with ciliary calcium or cAMP sensors is necessary to establish this paradigm. We propose that downstream signaling nodes commonly activated by multiple pro-adipogenic, ciliary signaling pathways may be particularly enticing therapeutic targets to increase the adipogenic potential of pre-adipocytes.

## Author Contributions

KH wrote the manuscript.

## Conflict of Interest

The author declares that the research was conducted in the absence of any commercial or financial relationships that could be construed as a potential conflict of interest.

## Publisher’s Note

All claims expressed in this article are solely those of the authors and do not necessarily represent those of their affiliated organizations, or those of the publisher, the editors and the reviewers. Any product that may be evaluated in this article, or claim that may be made by its manufacturer, is not guaranteed or endorsed by the publisher.
